# Supplementary material for: Can 17 hydroxyprogesterone caproate (17P) decrease preterm deliveries in patients with a history of PMC or pPROM?
Source: PLoS One. 2022 May 12;17(5):e0268397. doi: 10.1371/journal.pone.0268397 (PMC9098016; doi:10.1371/journal.pone.0268397)
Supplement: S1 Table — (DOCX) [file pone.0268397.s001.docx]

Supplementary table 1: Inclusion and exclusion criteria for the study

| **Inclusion criteria** | **Exclusion criteria** |
| --- | --- |
| Singleton pregnancies | Multiple gestations |
| Patients with 2 consecutive deliveries in our institution with the first delivery at < 37w GA | Preterm induction of labor due to PET, IUGR, NRFHR, fear of uterine rupture, suspected placental abruption or IUFD |
|  | Spontaneous preterm birth due to placental abruption |
|  | Patients administered with other modes of progesterone in subsequent pregnancy (Vaginal, PO) |
|  | Cerclage in any of the pregnancies |
|  | Pessary in any of the pregnancies |
